# Impact of baseline culture conditions of cancer organoids when determining therapeutic response and tumor heterogeneity

**DOI:** 10.1038/s41598-022-08937-z

**Published:** 2022-03-25

**Authors:** Rebecca A. DeStefanis, Jeremy D. Kratz, Autumn M. Olson, Aishwarya Sunil, Alyssa K. DeZeeuw, Amani A. Gillette, Gioia C. Sha, Katherine A. Johnson, Cheri A. Pasch, Linda Clipson, Melissa C. Skala, Dustin A. Deming

**Affiliations:** 1 Division of Hematology, Medical Oncology, and Palliative Care, Department of Medicine, University of Wisconsin School of Medicine and Public Health, University of Wisconsin-Madison, 1111 Highland Ave, 6507 WIMR2, Madison, WI 53705 USA; 2 University of Wisconsin Carbone Cancer Center, Madison, WI USA; 3 Department of Biomedical Engineering, University of Wisconsin-Madison, Madison, WI USA; 4 McArdle Laboratory for Cancer Research, Department of Oncology, University of Wisconsin-Madison, Madison, WI USA; 5 Morgridge Institute for Research, Madison, WI USA

**Keywords:** Cancer models, Cancer, Tissue culture, Gastrointestinal models

## Abstract

Representative models are needed to screen new therapies for patients with cancer. Cancer organoids are a leap forward as a culture model that faithfully represents the disease. Mouse-derived cancer organoids (MDCOs) are becoming increasingly popular, however there has yet to be a standardized method to assess therapeutic response and identify subpopulation heterogeneity. There are multiple factors unique to organoid culture that could affect how therapeutic response and MDCO heterogeneity are assessed. Here we describe an analysis of nearly 3500 individual MDCOs where individual organoid morphologic tracking was performed. Change in MDCO diameter was assessed in the presence of control media or targeted therapies. Individual organoid tracking was identified to be more sensitive to treatment response than well-level assessment. The impact of different generations of mice of the same genotype, different regions of the colon, and organoid specific characteristics including baseline size, passage number, plating density, and location within the matrix were examined. Only the starting size of the MDCO altered the subsequent growth. These results were corroborated using ~ 1700 patient-derived cancer organoids (PDCOs) isolated from 19 patients. Here we establish organoid culture parameters for individual organoid morphologic tracking to determine therapeutic response and growth/response heterogeneity for translational studies.

## Introduction

Colorectal cancer (CRC) is the second leading cause of cancer related deaths in the United States and is estimated to cause approximately 53,000 deaths in 2021^1,2^. Clinical treatments for metastatic CRC have shifted drastically over the past decade owing to a greater understanding of how the molecular profile of a cancer can guide clinical care strategies. More specifically, precision-guided approaches, such as anti-epidermal growth factor receptor inhibitors and immune checkpoint inhibitors are used for *KRAS/NRAS/BRAF* wild-type and mismatch repair deficient CRCs, respectively^3^. Despite these clinical advancements, preclinical models available to identify and study potential therapeutic strategies for these and other emerging subtypes of CRC remain few. Further, models such as historical 2-dimensional cell culture are limited in their ability to faithfully represent the disease^4–6^.

Cancer organoid cultures continue to be a major advance for studying therapeutic strategies. Organoid cultures are three-dimensional (3D) cell cultures that can be isolated from patient or mouse tumors and are grown in extracellular matrices, such as Matrigel or collagen. Compared to traditional 2D immortalized cell lines, cancer organoids better recapitulate the tumor from which they were derived, both morphologically and molecularly^7–21^. Historically, it has been difficult to establish 2D cell lines from more common cancer types in part owing to their inability to adhere to plastic^22,23^. The development of cancer organoids has significantly increased our ability to establish patient specific cultures across cancer types.

With this advancement in culture techniques, research groups have utilized both mouse and patient-derived cancer organoids to test pre-clinical hypothesis-driven combination therapies and to identify novel therapeutic strategies with high-throughput drug screens^8,24–28^. Given their 3D structure, traditional therapeutic response assessments used for 2D cultures are not directly transferable to organoid cultures. For this reason, multiple methods have been developed or adapted to assess response in these cancer organoids. Most assays measure metabolic activity of the cells at the well-level. One major pitfall to this method is its inability to evaluate the heterogeneity within a culture because these assays measure the gross response of the population within a given well. Additionally, longitudinal monitoring is difficult as these assays are very sensitive to baseline plating of the organoids, which is more challenging to control than with 2D cultures. To address this, our group has developed assays that measure the change in size or diameter of individual organoids over the course of the study. This method allows for the examination of an individual response of an organoid in addition to a population response^10,18,19,29–33^.

With the goal of using these organoid cultures as preclinical models to identify and confirm new therapeutic strategies, it is important to understand whether certain culture conditions affect growth and therapeutic response. Besides studies of media and supplements, limited data exists regarding the effect of baseline culture conditions on growth and response in these heterogeneous organoid cultures^26,29,34^. Several groups have investigated how different factors added to the media affect the maintenance and development of different organoid models^26,27,35–39^. However, no prior studies have performed a comprehensive analysis to examine how factors other than the media conditions alter growth or response of individual organoids. Here, we have evaluated a mouse-derived cancer organoid (MDCO) model developed by our group and several patient-derived cancer organoids (PDCOs) to address this knowledge gap. These MDCOs and PDCOs were established and analyzed over a 5-year period, enabling a direct comparison of data collected years apart from independent cultures. Specifically, we investigated if significant variation was seen between MDCOs derived from different mice of the same genotype or regions of the colon. Additionally, we assessed culture conditions including baseline size or location within the Matrigel droplet of individual MDCOs, the passage number of the line, and the density of the culture to determine whether these baseline conditions affect growth and response (Table 1). We further extended these findings by examining these culture conditions in the panel of 22 unique PDCOs isolated from 19 patients.

**Table 1:** Number of individual organoids used in the growth and response organoid characteristic analyses.

Organoid characteristics total # of organoids)	Figure	Inhibitors assessed	N of organoids
**MDCO growth**			
Matrigel variation	Suppl. S1a	NA	1791
Change point analysis	2	NA	994
Mouse	3a	NA	1001
Tumor location	3b	NA	855
Passage number	3e	NA	958
Location within Matrigel droplet	4b	NA	554
Density	4d	NA	3580
Density (prospective)	4g	NA	362
**MDCO response**			
Matrigel variation	Suppl. S1b	Vistusertib, copanlisib, sapanisertib	1791
Mouse	3c	Vistusertib, copanlisib, sapanisertib	1791
Tumor location	3d	Vistusertib, copanlisib, sapanisertib	1514
Passage number	3f	Vistusertib, copanlisib, sapanisertib	1691
Location within Matrigel droplet	4c	Vistusertib, copanlisib, sapanisertib	932
Density	4e	Vistusertib, copanlisib, sapanisertib	4771
Density (prospective)	4h	Vistusertib, copanlisib, sapanisertib	498
**PDCO growth**			
Baseline size	6b	NA	1740
Relative passage number	6c	NA	1740
Density	6d	NA	878
Location within Matrigel droplet	6e	NA	431
**MDCO growth (CPA applied)**			
Matrigel variation	Suppl. S1c	NA	834
Mouse	Suppl. S2a	NA	834
Tumor location	Suppl. S2b	NA	734
Passage number	Suppl. S3a	NA	817
Location within Matrigel droplet	Suppl. S4a	NA	501
Density	Suppl. S5a	NA	3277
**MDCO response (CPA applied)**			
Matrigel variation	Suppl. S1d	Vistusertib, copanlisib, sapanisertib	1598
Mouse	Suppl. S2c	Vistusertib, copanlisib, sapanisertib	1598
Tumor location	Suppl. S2d	Vistusertib, copanlisib, sapanisertib	1380
Passage number	Suppl. S3b	Vistusertib, copanlisib, sapanisertib	1512
Location within Matrigel droplet	Suppl. S4b	Vistusertib, copanlisib, sapanisertib	851
Density	Suppl. S5b	Vistusertib, copanlisib, sapanisertib	4685

The number (N) of organoids used in each organoid characteristic analysis is listed, along with the corresponding figure number. For the response analyses, all PI3K pathway inhibitors used are listed.

## Results

### Cancer organoids grow heterogeneously within a culture

**Figure 1:**
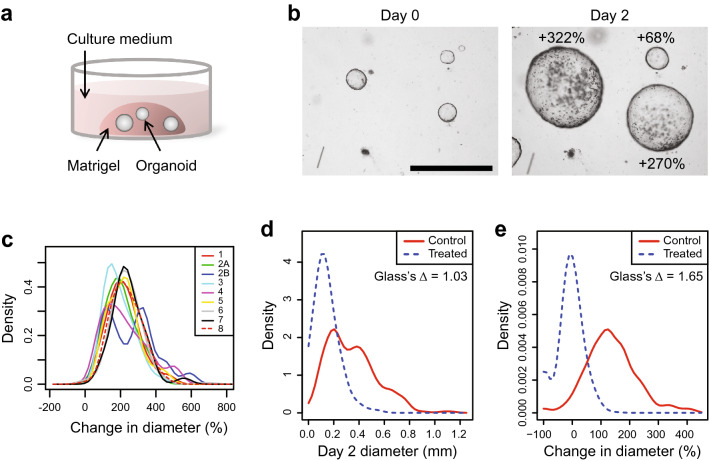
Mouse derived cancer organoids grow heterogeneously within a culture. (**a**) Graphic illustrating how MDCOs isolated from *Fc*^*1*^* Apc*^*fl/*+^ *Pik3ca*^*H1047R*^ mice are cultured within a Matrigel matrix, with feeding media overlayed. For therapeutic studies, overlayed feeding media is replaced with new media containing drug. (**b**) Kernel density plot comparing the growth of MDCOs at the population level derived from 8 different mice of the same genotype. Each line indicates the growth of MDCOs in feeding media derived from one mouse. Note that mouse 2 had two MDCO lines derived from two distinct tumors (line 2A and 2B). (**c**) Representative image of the heterogeneous growth rates of MDCOs within a culture over 48 h. (**d**) Density plot comparing the growth and response of MDCOs using only the final diameter (mm) after 48 h. This analysis represents a well level analysis where MDCOs are only evaluated on the final day of a study. Effect size was calculated using Glass’s delta to compare the treatment group to the control group. (**e**) Kernel density plot comparing the growth and response of MDCOs using their percent change in diameter over the 48-h incubation. This analysis examines the MDCOs on an individual level to determine how individual MDCOs change in diameter over the course of a study. Effect size was calculated using Glass’s delta to compare the treatment group to the control group. The studies used in (**d**) and (**e**) include MDCOs treated with normal feeding media (Control) or copanlisib (200 nmol/L) (Treated) for 48 h. Representative images from (**c**) are shown at the same magnification. Size bar, 1 mm.

We have previously shown the use of MDCOs derived from *Fc*^*1*^*Apc*^*fl/*+^ *Pik3ca*^*H1047R*^ (APPK) transgenic mouse CRCs as a model to examine potential therapeutic strategies, specifically those targeting the PI3K pathway^40^. Here we examine MDCOs from this model to assess the effects of baseline culture conditions on growth and response to targeted therapies. Briefly, these MDCOs are cultured in the extracellular matrix Matrigel and plated in droplets with media overlayed on top (Fig. 1a). The diameter of each organoid was measured at baseline and after 48 h with care taken to track the change in growth of each individual organoid over time. Within each culture, differential organoid growth is observed (Fig. 1b). This heterogeneity is maintained across numerous cultures derived from different mice and yields similar distributions of the change in organoid diameter (Fig. 1c).

Multiple methods have been developed to assess therapeutic response using 3D organoids. Most of those methods examine the organoids on a whole-well basis to examine the population response, usually on the final day of analysis. Metabolic assays are grossly affected by organoid plating which is much more challenging to control than in classic 2D cultures. Even in the setting where each individual organoid diameter is examined, the 48-h time point measurements alone lack sensitivity to detect treatment response (Fig. 1d) compared to changes in diameter of individual organoids over 48 h, likely due to differences in baseline organoid sizes (Fig. 1e). Additionally, a larger effect size is observed between the treatment and control for the change in diameter versus the 48-h time point alone (Glass’s delta (GΔ) values 1.65 vs 1.03, respectively)^41^. This ability to examine response on the single organoid level over time results in greater sensitivity to treatment response. To exclude the possibility that Matrigel variability might be influencing growth and drug response we compared the growth and response of MDCOs to the year the original treatment study was conducted. There was not a statistical t difference between earlier years (2016 and 2017) and later years (2019 and 2020) in growth and response (Growth: GΔ 2016: 0.2, 2017: 0.3, 2019: 0.1, 2020: 0.2, Response: GΔ 2016: 0.4, 2017: 0.4, 2019: < 0.1, 2020: 0.3) (Suppl. Fig. S1). With Matrigel variability excluded as a potential variable, we sought to examine whether any of the heterogeneity observed could be due to organoid culture conditions rather than individual organoid biology. Further investigations into how changes in the culture conditions could alter organoid growth were performed.

### Growth rate of MDCOs change as a function of their starting size

**Figure 2:**
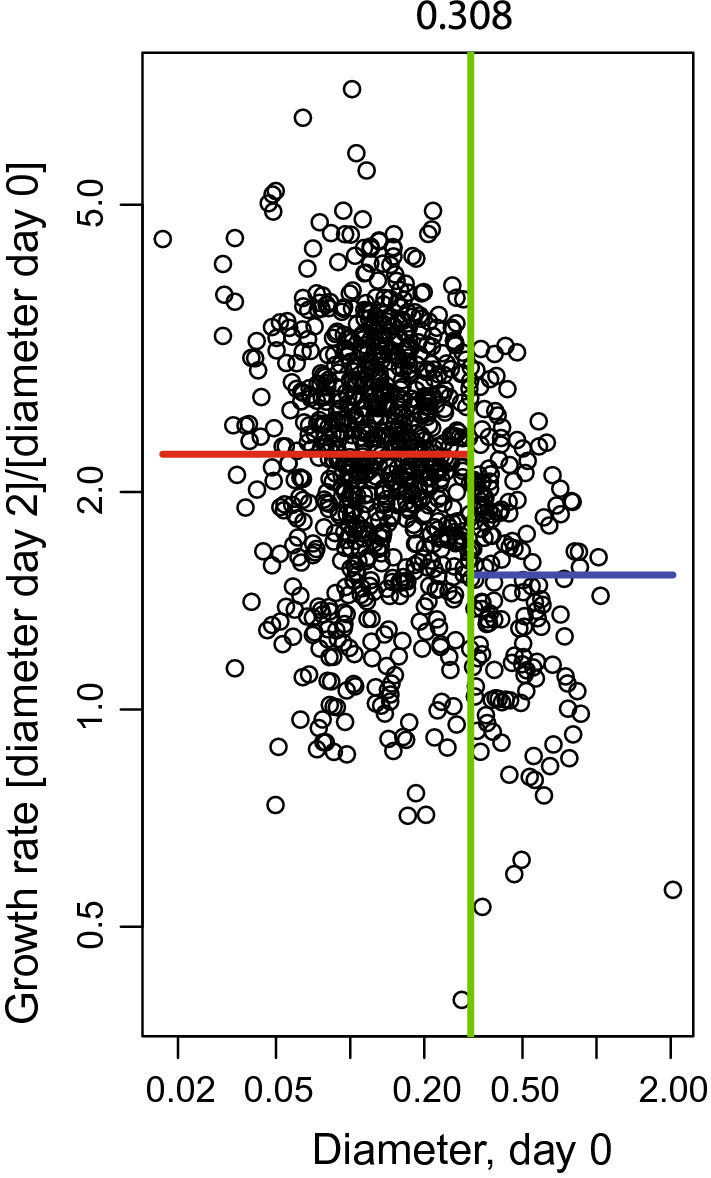
A standard change point analysis demonstrates that the growth rates of the MDCOs vary as a function of their size. A change point analysis at 308 µm was determined using all control APPK MDCOs. When the change point analysis was applied to all studies, only MDCOs < 308 µm on day 0 were used in the analyses (Suppl. Figs. S2–S6). The green line indicates the calculated change point value. The red line indicates the geometric mean of the data points that are lower than the determined change point value. The blue line indicates the geometric mean of the data points that are greater than the determined change point value (n = 994).

Previously, using a standard change point analysis (CPA), we reported that APPK MDCOs < 373 µm at baseline had a similar growth rate. However, MDCOs that were ≥ 373 µm had a reduction in growth rate and were therefore excluded from analysis^40^. We reapplied this change point analysis to include all of the control MDCOs in this analysis (n = 1019), which include those from our previously published work, and found that with this larger dataset, 308 µm is the more accurate change point value. This indicates that MDCOs that are ≥ 308 µm at baseline should be excluded from downstream analyses because the growth rate changes as a function of their size (Fig. 2). We applied this cutoff to our analyses and found that < 450 individual MDCOs were ≥ 308 µm at the beginning of these studies. These MDCOs encompassed only ~ 12% of all MDCOs used in subsequent organoid characteristic analyses. Approximately 40% of the MDCOs were controls and 60% were MDCOs treated with therapeutic agents. MDCOs with baseline sizes above the change point were excluded from subsequent supplemental analyses (Suppl. Figs. S2–S6).

### MDCOs derived from different mice of the same genotype and from different regions of the colon do not vary significantly in growth or drug response

**Figure 3:**
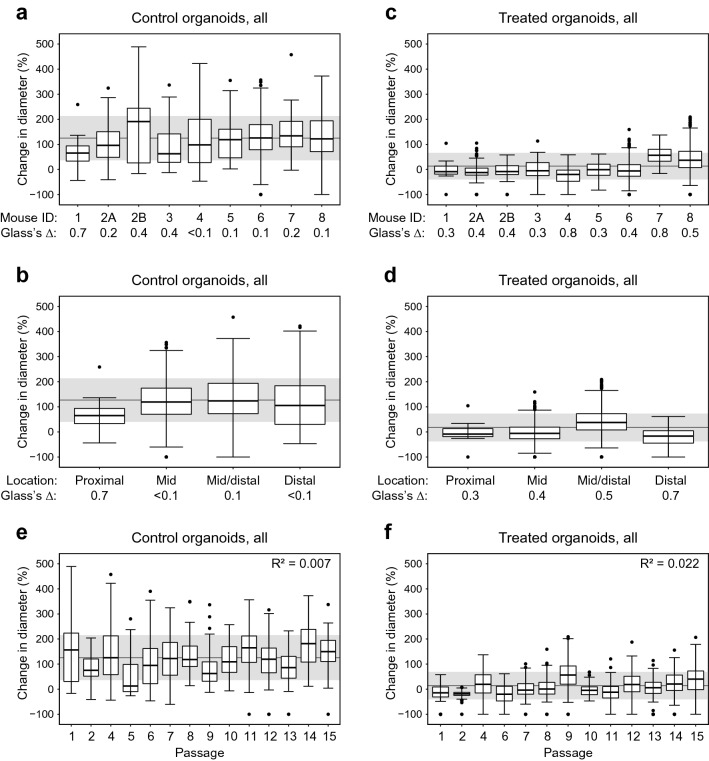
MDCOs derived from different mice of the same genotype or from different regions of the colon and passage number do not vary significantly in growth or drug response. Box and whisker plots displaying control organoids from (**a**) different mice of the same genotype and (**b**) tumors isolated from different regions of the colon. Box and whisker plots displaying treated organoids (**c**) derived from different mice of the same genotype and (**d**) tumors isolated from different regions of the colon. Each number represents a different mouse (1: n = 25 and n = 9, 2a: n = 125 and n = 234, 2b: n = 21 and n = 43, 3: n = 61 and n = 49, 4: n = 76 and n = 85, 5: n = 30 and n = 31, 6: n = 269 and n = 539, 7: n = 38 and n = 46, 8: n = 356 and n = 755, growth and response, respectively). Note that the MDCO line derived from mouse 1 was isolated from a proximal colon tumor and mouse 2 had two MDCO lines derived from two different colon tumors (distal: n = 106 and n = 116, mid/distal: n = 394 and n = 801, mid: n = 330 and n = 588, prox: n = 25 and n = 9, growth and response, respectively). Closest to the small intestine is the proximal colon moving more distally to the mid colon and finally the distal colon. Note the proximal colon has a different gross histology than the mid or distal colon. Box and whisker plots displaying the percent change in diameter for (**e**) growth and (**f**) drug response of MDCOs from passages 1–15 (P1: n = 26 and n = 69, P2: n = 23 and n = 63, P3: n = 0, P4: n = 87 and n = 91, P5: n = 10 and n = 0, P6: n = 68 and n = 83, P7: n = 82 and n = 84, P8: n = 57 and n = 132, P9:, n = 101 and n = 268, P10: n = 44 and n = 56, P11: n = 98 and n = 156, P12: n = 90 and n = 115, P13: n = 96 and n = 223, P14: n = 104 and n = 201, P15: n = 82 and n = 150, growth and response, respectively). In all box and whisker plots the grey line indicates the population change in diameter mean while the grey shading indicates one standard deviation above and below the population mean. Effect size was calculated using Glass’s delta to compare each mouse or tumor to the appropriate population mean.

One important feature of transgenic mouse models is the ability to generate mice within litters and across generations that have identical activation of transgenes and near identical genetic backgrounds. For this reason, we can isolate new or additional APPK MDCO lines from different mice of the same genotype both within a litter and across generations. To confirm that MDCO lines from different mice of the same genotype have similar growth distributions, a total of 8 different MDCO lines from 8 different cancers were isolated from 7 mice, two lines being isolated from two different tumors in one mouse. Minimal variation was seen across the growth of these MDCO lines as the GΔ values were less than 1.0 when each mouse was compared to the population (1: 0.69, 2a: 0.23, 2b: 0.40, 3: 0.38, 4: 0.01, 5: 0.06, 6: 0.08, 7: 0.22, 8: 0.09) and the majority of the individual MDCOs were within 1 standard deviation (SD) of the population mean (PM) (125 ± 88%) (Fig. 3a).

The *Fabp1-Cre* drives Cre-recombinase expression and subsequent constitutive recombination of transgenes in the epithelial portion of the distal small intestine and the large intestine. Therefore, tumor formation can occur anywhere in those regions of recombination. Tumors are isolated from multiple regions of the large intestine to establish APPK MDCO lines. APPK MDCO lines were derived from tumors in the proximal colon, which is closest to the stomach, the mid colon, mid/distal colon, and distal colon. It is important to note that the gross histology of the proximal colon is different from that of the mid and distal colon. Additionally, due to this difference in gross histology, only one mouse line (mouse 1) was found to have been isolated from the proximal colon. No significant variation was observed in the growth of the MDCO lines due to their original tumor location (GΔ distal: 0.01, mid/distal: 0.1, mid: 0.00, prox: 0.69, PM: 127, ± 87%) (Fig. 3b). MDCOs that were ≥ 308 µm at baseline were then removed from these analyses, based on the CPA from Fig. 2. We still found that growth was not affected by which mouse the line was derived from or which region of the colon the tumor was from (Mouse GΔ 1: 0.65, 2a: 0.21, 2b: 0.57, 3: 0.15, 4: 0.16, 5: 0.01, 6: 0.01, 7: 0.24, 8: 0.01, 137 ± 88%; Tumor GΔ distal: 0.09, mid/distal: 0.03, mid: 0.03, prox: 0.12, PM: 138 ± 86%) (Suppl. Fig. S2a,b).

Both variation in the mouse from which the organoids were derived and original tumor location were evaluated as potential conditions that might alter treatment response, in this case to PI3K pathway inhibition. Work from our group, using APPK MDCOs, has demonstrated that dual MTORC1/2 inhibition is sufficient to induce a treatment response in *Pik3ca* mutant CRC. To assess therapeutic response, the overlayed medium was replaced with new medium containing drug, and the diameter of each organoid measured at baseline and after 48 h to track how individual MDCOs and the population of MDCOs respond to a given therapy^40,42^. These studies were used in this pooled analysis along with other studies that assessed the efficacy of PI3K pathway inhibitors. Altogether five PI3K pathway inhibitors with doses ranging from 5-500 nmol/L including those that inhibit both PI3K/MTOR (dactolisib, copanlisib), MTORC1 (everolimus), and both MTORC1/2 (vistusertib, sapanisertib) were used in subsequent analyses (Table 2).

**Table 2:** PI3K pathway inhibitors and doses of inhibitors used in organoid response characteristics analyses. Multiple PI3K pathway inhibitors were assessed in the organoid response characteristic analyses ranging from 5 to 500 nmol/L.

Inhibitor	Target	Doses (nmol/L)
Vistusertib	mTORC1/2	200, 300, 400, 500
Dactolisib	PI3K/mTOR	100, 200, 400
Copanlisib (BAY 80-6946)	PI3K/mTOR	5*, 10*, 100, 200, 400
Everolimus	mTORC1	100*, 200*, 400*
Sapanisertib	mTORC1/2	100, 200, 400

*Doses used only in UMAP analyses.

The responses of all MDCOs treated with a PI3K pathway inhibitor were grouped based on the mouse number and the original tumor location. No significant difference was seen across MDCOs separated by mouse number or tumor location (GΔ 1: 0.28, 2a: 0.44,2b: 0.41, 3: 0.27, 4: 0.83, 5: 0.34, 6: 0.38, 7: 0.79, 8: 0.52, PM: 13 ± 53% and GΔ distal: 0.28, mid/distal: 0.38, mid: 0.53, prox: 0.70, PM 18 ± 55%, respectively) (Fig. 3c,d). Interestingly a proportion of the individual MDCOs from mouse 1, which was the only MDCO line originally isolated from the proximal colon, fell outside of 1 SD from the PM both in the different mouse line assessment and original tumor location assessment (Fig. 3c,d). This could be due to the gross histological differences between the proximal colon and the rest of the colon, though more studies are needed to confirm this explanation. With the CPA applied, we observed similar results that indicated mouse number (GΔ 1: 0.29, 2a: 0.47,2b: 0.40, 3: 0.31, 4: 0.82, 5: 0.20, 6: 0.40, 7: 0.86, 8: 0.49, PM 15 ± 54%) and tumor location (GΔ distal: 0.29, mid/distal: 0.39, mid: 0.51, prox: 0.65, PM 19 ± 56%) do not affect the MDCO drug response (Suppl. Fig. S2c,d).

Overall, these data indicate that growth of MDCOs are not affected by being isolated from different mice of the same genotype or original tumor location. However, our studies suggest that MDCO lines isolated from the proximal colon versus mid or distal colon could have some variation in how they respond to treatment. These differences suggest that MDCOs derived from the proximal colon should be further investigated and not directly compared to MDCOs derived from the mid, mid/distal, or distal colon.

### Passage number of cultures does not affect growth or drug response of APPK MDCOs

Once it was established that there was no significant variation in growth or response at the mouse level, we sought to look further in depth at specific baseline culture conditions. We next evaluated if passage number influences growth or response of the APPK MDCOs. It is well established that in traditional 2D cell lines higher passaged cells can develop significant alterations in growth rates, and in how they respond to therapies, among other characteristics^4–6,43–45^. We analyzed MDCOs ranging from passage 1–15 across different lines from studies of PI3K pathway inhibitors. We observed that no significant variation was observed in growth as there was no correlation between growth and passage number (R^2^ = 0.007, PM 126 ± 89%) (Fig. 3e). Similar observations with response were seen in the treated MDCOs. The response of the majority of the treated MDCOs displayed no correlation with passage number (R^2^ = 0.022, PM 14 ± 54%) (Fig. 3f). We further excluded the MDCOs that were ≥ 308 µm as determined in Fig. 2 and observed that the majority of MDCOs’ did not correlate with passage number (R^2^ = 0.001, PM 138 ± 88% and R^2^ = 0.012, PM 16 ± 55%, respectively) (Suppl. Fig. S3). We did note at passage 5 that some of the MDCOs’ change in diameter fell below 1SD of the PM, however (Fig. 3e), the dataset was limited (n = 10 and n = 0) for growth and response assessment, respectively) making it difficult to accurately evaluate growth and response at this passage number.

### Location or density of the APPK MDCO within the in vitro matrix does not affect growth or response

**Figure 4:**
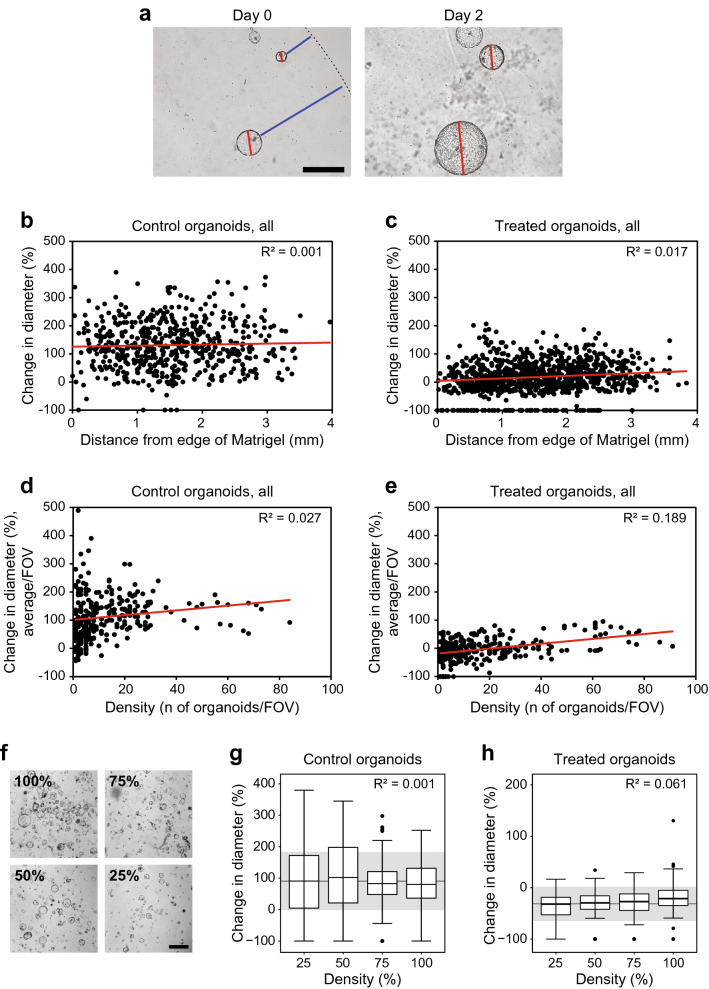
Location within the Matrigel matrix or density does not affect MDCO growth or drug response. (**a**) Representative images illustrating how location within the Matrigel matrix was done. Briefly, for each MDCO, the shortest distance (blue line) from the edge of the organoid to the edge of the Matrigel (dashed line) at the beginning of the study was measured using ImageJ. Scatter plots display individual organoids’ location within the Matrigel matrix vs their change in diameter in (**b**) growth and (**c**) drug response (n = 554 and n = 932, respectively). Linear trend lines are indicated in red. The R^2^ values displayed in the top right corner indicate that no significant correlation exists between the MDCOs change in diameter and location within the Matrigel matrix. Representative images from (**a**) are shown at the same magnification. Size bar, 500 µm. Scatter plots display if the density of MDCO cultures affect (**d**) growth or (**e**) drug response (n = 3580 and n = 4771, respectively). Density was determined by calculating the number of organoids per field of view (FOV) and was plotted against the average change in diameter per FOV. Linear trend lines are indicated in red. The R^2^ values displayed in the top right corner indicate the lack of significant correlation between change in diameter and the culture density. (**f**) Representative images illustrating starting density percentages used for the prospective analyses to validate observations seen in (**d**) and (**e**). Box and whisker plots displaying (**g**) control organoids and (**h**) treated organoids at different culture densities in the prospective study (n = 362 and n = 498). In all box and whisker plots the grey line indicates the population change in diameter mean while the grey shading indicates one standard deviation above and below the population mean. Representative images from (**f**) are shown at the same magnification. Size bar, 1 mm.

Unlike traditional 2D cultures, organoids are cultured within an extracellular matrix, such as Matrigel with feeding media overlayed (Fig. 1a). To assess therapeutic response, the overlayed feeding media is exchanged with fresh media containing drug. Given this significant difference in culture methods we aimed to confirm that the MDCOs both at the periphery and towards the center of the Matrigel droplet were growing and responding to the same extent. To assess the location of each MDCO within the Matrigel droplet, the shortest distance from an individual MDCO to the edge of the Matrigel droplet, as seen in the field of view (FOV) was measured. This was plotted against each MDCO’s percent change in diameter (Fig. 4a). The growth of the control MDCOs was not correlated with the location of the MDCOs within the Matrigel droplet (R^2^ = 0.001) (Fig. 4b). Similar observations were observed in the treatment response, as indicated by a low R^2^ value when comparing the treated MDCOs’ percent change in diameter to location within the Matrigel droplet (R^2^ = 0.017) (Fig. 4c). With the exclusion of the MDCOs that fell outside the appropriate starting size (i.e. excluding organoids ≥ 308 µm) no correlation was observed between location of the MDCO and growth (R^2^ = 0.001) or response to these small molecules (R^2^ = 0.017) (Suppl. Fig. S4).

We next evaluated whether density, which is equivalent to confluency in traditional 2D cultures, affects the growth or response of the MDCOs. To do this, the total number of MDCOs/field of view was calculated and compared to the average percent change in diameter of the MDCOs within that field of view. No association was seen between the density and the growth or response of the MDCOs (R^2^ = 0.027, R^2^ = 0.189, respectively) (Fig. 4d,e). Once the change point analysis was applied, similar observations of no correlation between density and growth or response were observed (R^2^ = 0.004, R^2^ = 0.108, respectively) (Suppl. Fig. S5).

This observation was confirmed in a prospective study where APPK MDCOs were plated at densities ranging from 25 to 100% (Fig. 4f) and allowed to mature for 24 h. Baseline 4 × brightfield images were taken and the overlayed media was exchanged with new media containing copanlisib (200 nmol/L) or control. After a 48-h incubation, the same MDCOs from day 0 were imaged again. The changes in diameter of control or treated MDCOs were compared across the different densities. We observed no correlation between growth and density of the MDCOs R^2^ = 0.0012, PM 91 ± 92%) (Fig. 4g). This was also observed in the copanlisib treated MDCOs (R^2^ = 0.0608, PM -31 ± 32.8%) (Fig. 4h).

### Multivariate analysis confirms baseline culture conditions do not affect growth or therapeutic response

**Figure 5:**
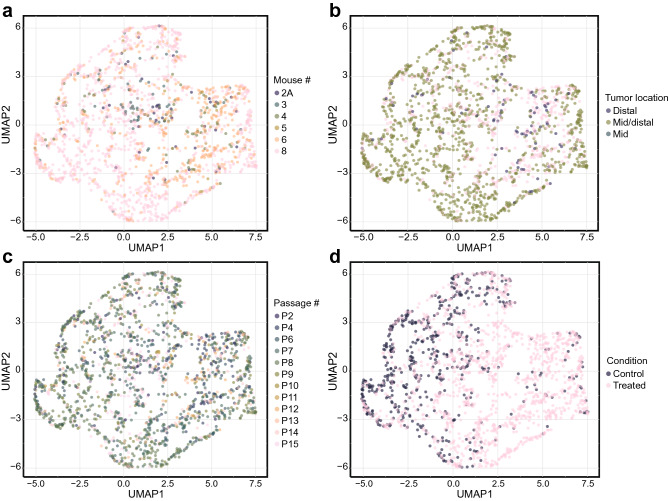
Multivariate analysis validates the finding that most baseline culture conditions do not affect growth or drug response. UMAP data-reduction examined four key variables (baseline diameter, distance to the edge of the Matrigel droplet, day 2 diameter, and percent change in diameter) to determine if baseline culture conditions caused clustering of individual MDCOs. UMAP visual representations showed that (**a**) MDCOs derived from different mice of the same genotype, (**b**) location of the original tumor within the colon, or (**c**) passage number did not cause clustering of MDCOs. Only (**d**) control and treated MDCOs showed any clear separation. (n = 1835 organoids).

Using a multivariate analysis, we confirmed that these baseline culture conditions do not cause unique clustering of organoids. A uniform manifold approximation and projection (UMAP) was used to examine four key variables for each individual organoid: baseline diameter, distance to the edge of the Matrigel droplet, day 2 diameter, and percent change in diameter over 48 h. This dimension reduction technique is similar to *t-*distributed stochastic neighbor embedding (*t*-SNE) and principal component analysis (PCA) but is faster and able to preserve more of the global data structure than other dimensional reduction techniques^46^. UMAP representations showed that no clear clustering of organoids occurred due to MDCOs being derived from different mice of the same genotype (Fig. 5a), location of the original tumor (Fig. 5b), or passage number of MDCOs (Fig. 5c). The only variable that showed clear clustering of MDCOs was whether the MDCO was treated with control media or a PI3K pathway inhibitor (Fig. 5d). This analysis was also done with the CPA applied. Similar results were found demonstrating that different mice (Suppl. Fig. S6a), location of the original tumor (Suppl. Fig. S6b), or passage number (Suppl. Fig. S6c) do not cause clear clustering of organoids. Only the treatment status of an organoid, i.e., if it was a control or treated organoid, showed clear clustering (Suppl. Fig. S6d).

### Growth of PDCOs is heterogeneous and not affected by baseline culture conditions

**Figure 6:**
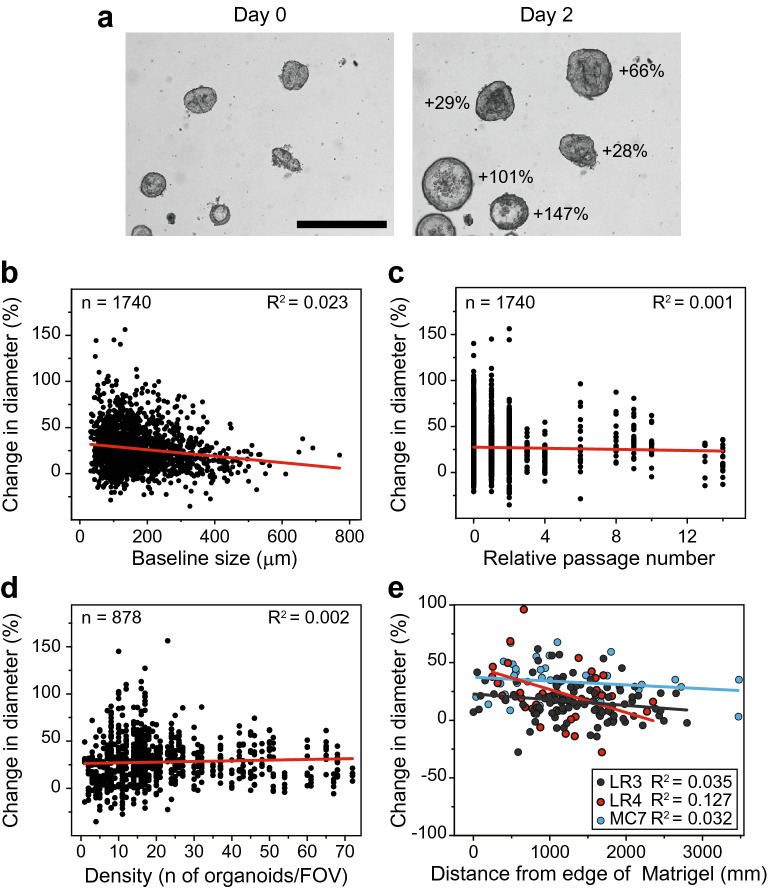
Growth of patient derived cancer organoids is heterogeneous and not affected by baseline culture conditions. Representative image of the heterogeneous growth rates of PDCOs within a culture over 48 h (**a**). Scatter plots display individual organoids’ baseline diameter size (µm) (**b**), relative passage number (**c**), and PDCOs per field of view (**d**) vs their change in diameter (n = 1740, n = 1740, n = 878, respectively). Scatter plots of three CRC PDCO lines location within the Matrigel droplet vs their changes in diameter (LR3 n = 213, LR4 n = 82, and MC7 n = 136). Glass’s delta was calculated to determine the effect size of the change in diameter vs location in the Matrigel droplet for each PDCO line. (**e**) Representative images from (**a**) are shown at the same magnification. Size bar, 500 µm.

Lastly, we applied these analyses to a panel of patient derived cancer organoids (PDCOs). Altogether we analyzed 1740 individual PDCOs across 22 lines isolated from 19 patients. Similar to the MDCOs, differential growth of the PDCOs was observed (Fig. 6a). Additionally, no correlation was found between the baseline PDCO size and the change in diameter (R^2^ = 0.023; Fig. 6b). Additionally, no change point value was found for any of the PDCO lines. Furthermore, it was determined that relative passage number and density of the PDCOs did not correlate with change in diameter (R^2^ = 0.001 and R^2^ = 0.002, respectively; Fig. 6c,d). Finally, using the LR4, LR5, and MC7 PDCOs analyses we found that the location of the PDCO within the Matrigel droplet did not correlate with growth (LR3 R^2^ = 0.035, LR4 R^2^ = 0.127, MC7 R^2^ = 0.032; Fig. 6e). Altogether, these data illustrate that PDCO growth is heterogeneous and not affected by the baseline size, relative passage number, density, or location within the Matrigel droplet.

## Discussion

Three-dimensional (3D) cancer organoids, derived from patient or mouse cancers, are becoming an increasingly popular model to identify and study novel combination therapies across many cancer types. Our group and others have shown that these in vitro models better recapitulate the genetic, morphological, and phenotypic characteristics of the cancers from which they were derived compared to traditional 2D immortalized cell lines^8–21^. One notable advantage to organoid cultures is their ability to be readily isolated from a small amount of tissue sample, such as surgical needle biopsies and only require ~ 20–30 organoids/condition to provide enough statistical power to test targeted therapies.

In many cancer types, including CRC, there is a growing appreciation for how the molecular profile of a tumor will affect therapeutic response^3^. Therefore, patient-derived cancer organoids provide an attractive model to predict patient response and guide clinical treatment decisions^10,19,21,29,30,33^. Alternatively, MDCOs are readily isolated from various transgenic mouse models across mutational profiles and diseases, providing a more controlled and high-throughput platform for screening potential therapies.

Given the recent development of cancer organoids, there have yet to be well-validated methods to determine response in these 3-dimentional cultures. Traditional 2D culture therapeutic response assessment techniques do not readily transfer to 3D organoid cultures as even basic culturing methods differ between 2 and 3D cultures. Many groups use methods that assess response on a well-by-well basis. Additionally, many assay rely on the addition of reagents that do not allow for longitudinal assessment^9,14,16,47–53^. These types of assays ignore many of the baseline characteristics of the organoids including heterogeneity within a culture. Some groups, including ours, evaluate the organoids on an individual basis using change in diameter or volume^10,29–32^. Not only is this a less invasive method to determine response, but individual organoids can be tracked repeatedly throughout a longer study and allows for capturing subpopulations (Fig. 1). This has the potential to identify resistant clones and mechanisms of resistance to therapies.

If these organoid cultures are to be used to identify potential novel therapeutic strategies, it is important to understand how the culture conditions might affect the assays. Previously our group has shown that MTORC1/2 inhibition is necessary for a response in *Apc* and *Pik3ca* mutant CRC using the APPK MDCOs^40^. We used this model in the context of PI3K pathway inhibition to determine if various culture conditions affect response. Notably, our MDCO lines, PDCO lines, and corresponding data were collected over the course over a 5-year period and from mice established across many generations and 19 individual patients. This allowed us to confirm that direct comparisons of data collected years apart from different cultures could be made.

We had previously observed and reported with a smaller cohort of MDCOs that those that were larger in size tended to grow at a slower rate^40^. With this larger cohort, which included those MDCOs originally assessed, we determined that APPK MDCOs ≥ 308 µm in diameter at baseline have a slower growth rate (Fig. 2). Excitingly, many of the results were better corroborated with the exclusion of MDCOs ≥ 308 µm (Suppl. Figs. S2–S6).

We then broadly examined if any variation was seen between cultures derived from different mice of the same genotype or tumors isolated from different regions of the large intestine. While all mice were the same genotype, it is important to determine if any significant variation was seen among different mice across generations or even from different regions within the large intestine as gross histology varies slightly throughout the intestine. Significant variation between different mice or different regions of the colon could indicate some underlying differences in the biology of these mice or large intestine. No significant difference in growth or response to these PI3K pathway inhibitors was found. However, some variation in response was seen in those treated MDCOs isolated from the proximal colon when compared to those from the other locations. The gross histological differences may account for some of the variation seen in these treated MDCOs (Fig. 3a–d). Altogether this data demonstrates that comparisons of growth and response can be made between MDCOs across several years of mouse colony propagation.

Once we established that growth and response of MDCOs can be reliably examined across generations of mice, we assessed the influence of MDCO culture conditions. It is well established that immortalized 2-dimentional cultures can have significant changes as they increase in passage number due to genetic and phenotypic drift. We confirmed that no significant difference in growth or response develop as MDCOs are maintained in culture, up to passage 15 (Fig. 3e,f). Further studies are needed to assess whether culture beyond 15 passages promotes a change in growth or response in MDCOs.

A unique aspect of MDCO cultures is their growth and maintenance in an ECM such as Matrigel. While other biomaterials such as collagen or synthetic matrices are available, Matrigel is widely utilized, cost effective, and is comprised of many ECM proteins, making it a robust matrix for organoid cultures^18^. Additionally, other groups are continuing to assess biomaterial alternatives. Regardless of the matrices used, these unique culture conditions may provide a physical challenge for nutrient and drug delivery to MDCOs in the centermost region of the Matrigel droplet. We sought to address this concern by measuring MDCOs’ location within the Matrigel droplet to compare the growth and response between MDCOs at the center and edge of the droplet. We evaluated MDCOs as far as 4 mm deep and found no correlation between MDCO location within the droplet and growth or response to PI3K pathway inhibitors. This demonstrates that the Matrigel droplet does not pose a significant barrier for nutrient and drug distribution of these small molecules (Fig. 4a–c). Matrigel may however pose a delivery challenge to larger therapies, such as antibodies, which warrants further investigations.

Arguably the most important feature of organoid cultures is their ability to grow as 3D organized structures. When traditional immortalized 2D cultures are grown as 3D structures, they are usually pelleted cells and not organized in a coherent fashion. This is potentially due to the result of two key but opposing features that 2D cell lines can possess: contact inhibition and overgrowth. In both cases, proliferation rates are significantly altered with confluency, which can affect therapeutic response^4–6,43–45^. We observed that neither growth nor response was altered due to the density of the culture (number of organoids/FOV). This was prospectively confirmed with an MDCO line cultured to its greatest density and diluted down to 25% of its highest density illustrating that MDCOs at different densities can be compared (Fig. 4d–h). Additionally, this demonstrates that MDCO cultures are more cancer-like, compared to many 2D cultures, in their ability to propagate without contact inhibition.

Using the dimension reduction technique UMAP as a comprehensive, simultaneous analysis, we demonstrated that MDCOs only separate based on whether they were treated with an inhibitor or control media and not many of the baseline culture conditions assessed (Fig. 5).

Finally, we extended this work to 22 PDCO lines isolated from 19 CRC patients, which possess significantly more genomic heterogeneity. We found significant heterogeneity in growth across these 22 PDCO lines. Interestingly none of the baseline culture conditions, including baseline size affected the growth of the PDCOs (Fig. 6). While further studies are warranted, we hypothesize that these conclusions would apply to other epithelial cancer organoid models due to their similarities in culturing^10,54^. Further work should determine if these results hold true in other newly established organoid models of other diseases.

Altogether we demonstrate that many baseline culture conditions do not affect the growth or response to small molecules of CRC MDCOs or the growth of CRC PDCOs. Additionally, our work demonstrates the importance of examining response at an individual organoid level as opposed to the population or well level in order to capture the heterogeneity of a culture. These studies bring to light the importance of understanding potentially confounding features to any assay, particularly drug assessment in 3D organoid models. This work also now sets the stage for further investigation into biologic differences within tumors using individual organoid analyses to better understand the underpinnings of tumor heterogeneity in cancer development and clonal evolution in response to anti-cancer therapeutics.

## Methods

### Cell isolation and organoid culture

All animal studies were performed adhering to approved protocols by the Institutional Animal Care and Use Committee at the University of Wisconsin (Madison, WI) following the guidelines of the American Association for the Assessment and Accreditation of Laboratory Animal Care International. This study is reported in accordance with ARRIVE guidelines. *Apc*^*fl/fl*^ mice (B6.Cg-*Apc*^*tm2Rak*^; NCL Mouse Repository; Strain number 01xAA), *Pik3ca*^*H047R*^ mice (FVB.129S6 Gt(ROSA)26Sortm1(Pik3ca*H1047R)Egan/J; The Jackson Laboratory; Stock Number 016977) and *Fc*^*2*^ mice [FVB/N-Tg(Fapb1-Cre)1Jig; NCI Mouse Repository; Strain number 01XD8] were used to generate *Fc*^*1*^*Apc*^*fl/*+^*Pik3ca*^*H1047R*^ mice and these mice were genotyped as previously described^55,56^. Colorectal cancer cells were isolated from *Fc*^*1*^* Apc*^*fl/*+^ *Pik3ca*^*H1047R*^ (APPK) mice and cultured in Matrigel (Corning, cat #75796-276) as previously described^42^. Fourteen different lots of Matrigel were purchased throughout the 5 years data was collected.

### Patient derived cancer cell isolation and organoid culture

All studies were approved by the Institutional Review Board (IRB) of the University of Wisconsin Madison Health Sciences IRB, with informed consent obtained from subjects through the University of Wisconsin Molecular Tumor Board Registry (Madison, WI) (UW IRB#2014-1370) or UW Translational Science BioCore (UW IRB#2016-0934). All methods were performed in accordance with the relevant guidelines and regulations. Tissue was obtained from needle, endoscopic biopsies or primary resections and processed as previously described^10^. These samples were named for their disease type: locally advanced (L), metastatic (M), rectal (R), and colon (C).

### Pharmacologic agents

The PI3K pathway inhibitors vistusertib (HY-15247, MedChem Express), everolimus (E-4040, LC Laboratories), dactolisib (N-4288, LC Laboratories), and sapanisertib (I-3344, LC Laboratories), were dissolved in DMSO to a 10 mmol/L stock concentration. Copanlisib (HY-15346, MedChem Express) was dissolved in 10 mmol/L TFA/DMSO to a 5 mmol/L stock concentration. Described inhibitors were diluted in fresh media at concentrations ranging from 5 nmol/L to 500 nmol/L for diameter studies.

### Baseline culture conditions analysis

Organoids were plated in 24-well tissue culture plates and allowed to mature for 24–96 h. Passages between 1 and 15 were used for the described MDCO studies. Relative passages between 0 and 34 were used for the described PDCO studies. Relative passage number for the PDCOs was defined by the current experiment passage number minus the first experiment used in the series for a given line. Prior to treatment, baseline 4 × images were taken on a Nikon Ti-S inverted microscope. After imaging, overlayed feeding media was replaced with fresh feeding media containing drug. Following 48 h of incubation at 37 °C and 5% CO_2_, posttreatment images were taken. All images were analyzed using ImageJ, measuring the longest diameter of each organoid and the shortest distance from the organoid edge to the Matrigel edge. In each study, metrics of baseline organoid size, passage number/relative passage number, plating density, location within the Matrigel droplet, and where appropriate, the mouse identification number were collected for analysis.

For studies examining differences in dilutions, APPK MDCOs were cultured to their highest density and diluted to 75%, 50%, and 25% of the highest density. Cell suspensions were then plated in a 1:1 ratio with Matrigel as previously described. Response was assessed by exchanging feeding media with fresh media containing copanlisib (200 nmol/L) or control and diameter studies continued as previously described^40^.

### UMAP analysis

Clustering of organoids across all mice and treatment conditions was represented using Uniform Manifold Approximation and Projection (UMAP) with the UMAP package in Python v3.7^46^. UMAP dimensionality reduction was performed on four key variables (Day 0 Diameter, Distance to Edge, Day 2 Diameter, Percent Change in Diameter) for projection in 2D space. Organoids without one of these measurements were removed from the analysis, which left 1835 organoids included in the UMAP. The following parameters were used for UMAP visualizations: “n _neighbors”: 200; “min_dist”: 0.2, “metric”: cosine, “n_components”: 2. The generated UMAP data frame was then merged with the original input data frame for visualization in R (R-studio v.1.4).

### Statistical analysis

A change point analysis is a statistical analysis used to determine if a change in ordered data has occurred. A change point analysis was conducted to assess the impact of baseline size^57^. The percent change in organoid diameter over 48 h was used to analyze growth and treatment response across baseline culture conditions. Kernel density plots using the *sm* package, Tukey’s boxplots, and scatter plots were generated using the *ggplot2* package and R software^58–60^. Glass’s delta was used to calculated treatment effect size^41^. For continuous variables, the glass’s delta of each variable was calculated against the population of that variable.

### Ethical approval

This study is reported in accordance with ARRIVE guidelines.

## Supplementary Information

Supplementary Information.
