# Supplementary material for: Impact of baseline culture conditions of cancer organoids when determining therapeutic response and tumor heterogeneity
Source: Sci Rep. 2022 Mar 25;12:5205. doi: 10.1038/s41598-022-08937-z (PMC8956720; doi:10.1038/s41598-022-08937-z)
Supplement: Supplementary file 1 — Supplementary Information. [file 41598_2022_8937_MOESM1_ESM.pdf]

Supplementary Table S1. Summary of clinical characteristics of patient-derived cancer organoids. Samples were named for their disease type: locally advanced (L), metastatic (M), rectal (R), and colon (C).

Name	Histology	Primary tumor	Site of tissue	Tissue sampling
LC1	Adenocarcinoma	Colon	Colon	Surgical resection
LC2	Adenocarcinoma	Colon	Colon (cecum)	Surgical resection
LR1A	Adenocarcinoma	Rectum	Rectum	Surgical resection
LR1B	Adenocarcinoma	Rectum	Rectum	Surgical resection
LR2	Adenocarcinoma	Rectum	Rectum	Surgical resection
LR3	Adenocarcinoma	Rectum	Rectum	Endoscopic biopsy
LR4	Adenocarcinoma	Rectum	Rectum	Endoscopic biopsy
LR5	Adenocarcinoma	Rectum	Rectum	Endoscopic biopsy
MC1	Adenocarcinoma	Colon	Liver	Surgical resection
MC2	Adenocarcinoma	Colon	Colon	Surgical resection
MC3A	Adenocarcinoma	Colon	Colon	Endoscopic biopsy
MC4B	Adenocarcinoma	Colon	Colon	Surgical resection
MC7	Adenocarcinoma	Colon	Colon	Endoscopic biopsy
MR1	Mucinous adenocarcinoma	Rectum	Peritoneum	Surgical resection
MR2	Adenocarcinoma	Rectum	Rectum	Endoscopic biopsy
MR3	Adenocarcinoma	Rectum	Lung	Needle biopsy
MR4	Adenocarcinoma	Rectum	Liver	Surgical resection
MR5	Mucinous adenocarcinoma	Rectum	Omentum	Surgical resection
MR6	Adenocarcinoma	Rectum	Liver	Surgical resection
MR7	Adenocarcinoma	Rectum	Liver	Needle biopsy

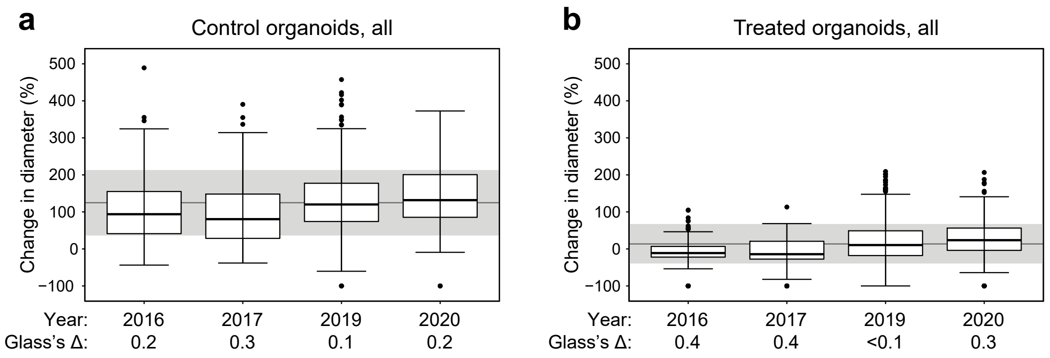

Supplementary Figure 1. Matrigel variability does not affect growth or response of MDCOs. Box and whisker plots displaying the (a) growth and (b) response of MDCOs against the year the experiment was originally completed. Each number represents a different year (2016: n = 171 and n = 286, 2017: n = 124 and n = 93, 2019: n = 383 and n = 879, 2020: n = 327and n = 533, growth and response respectively). Glass's delta was calculated for each year compared to the population mean.

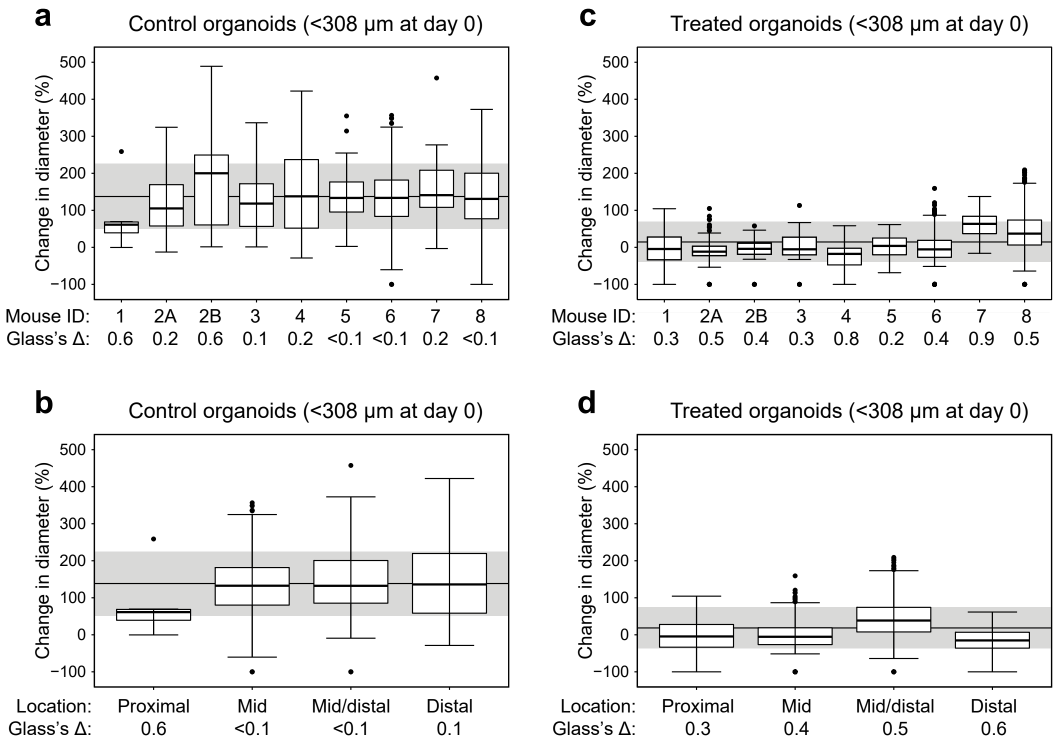

Supplementary Figure S2. Effect of derivation of MDCOs from different mice of the same genotype or from different regions of the colon with change point analysis applied. MDCOs with a starting diameter of ≥308µm were removed from these analyses. Box and whisker plots displaying (a) control organoids and (c) treated organoids derived from different mice of the same genotype (1: n = 6 and n = 4, 2a: n = 85 and n = 187, 2b: n = 15 and n = 33, 3: n = 37 and n = 29, 4: n = 60 and n = 72, 5: n = 23 and n = 28, 6: n = 249 and n = 507, 7: n = 32 and n = 39, 8: n = 327 and n = 701, growth and response, respectively). Box and whisker plots displaying (b) control organoids and (d) treated organoids derived from tumors isolated from different regions of the large intestine (distal: n = 83 and n = 100, mid/distal: n = 359 and n = 740, mid: n = 286 and n = 536, prox: n = 6 and n = 4, growth and response, respectively). Note that the gross histology of the proximal colon is different from the mid and distal colon. For all box and whisker plots the grey line is the population mean with the grey shading indicating ±1 standard deviation from the mean. Effect size was calculated using Glass's delta to compare individual mice or tumor locations to the population mean.

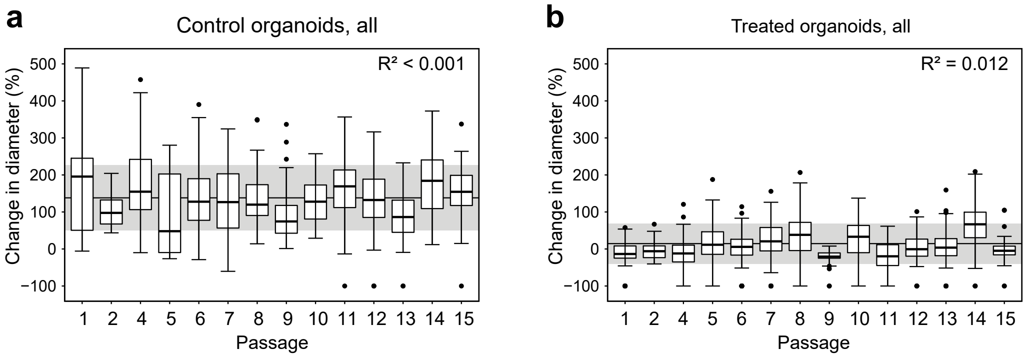

Supplementary Figure S3. The passage number of MDCOs does not affect growth or drug response when change point is applied. Box and whisker plots demonstrating (a) control organoids and (b) treated organoids at different passages (P1: n =20 and n = 51, P2: n = 16 and n = 46, P3: n = 0, P4: n = 63 and n = 70, P5: n = 6 and n = 0, P6: n = 43 and n = 75, P7: n = 71 and n = 71, P8: n = 55 and n = 126, P9: n = 74 and n = 234, P10: n = 38 and n = 44, P11: n = 92 and n = 151, P12: n = 77 and n = 95, P13: n = 85 and n = 209, P14: n = 99 and n = 198, P15: n = 78 and n = 142, growth and response, respectively). Passages range from 1-15 from isolation. The change point was applied and all MDCOs with a starting diameter of ≥308 µm were removed from these analyses. Note that passage 5 in the treated MDCOs only had n = 1. For all plots, the grey line is the population mean while the grey shading indicates ± 1 standard deviation from the mean.

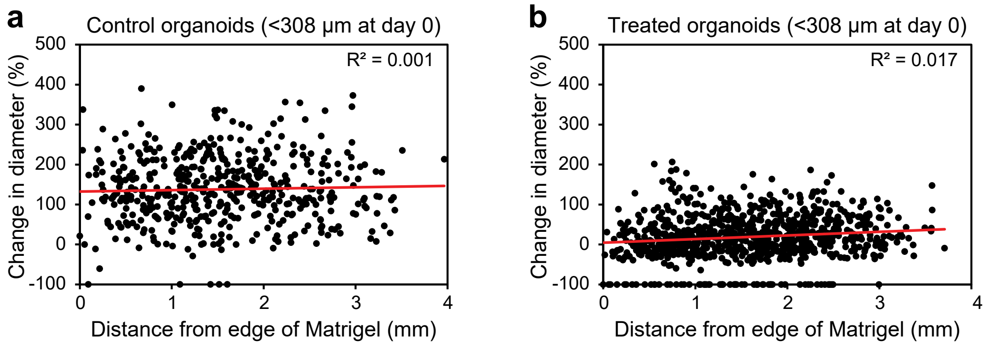

Supplementary Figure S4. Location of the MDCOs within the Matrigel matrix does not affect growth or drug response with the change point applied. Scatter plots display individual (a) control or (b) treated MDCOs distance from the edge of the Matrigel matrix (mm) plotted against its change in diameter n = 501 and n = 851, respectively). Change point analysis was applied and only MDCOs with a starting diameter size of <308 µm were included for analyses. The linear trend lines are indicated in red. Correlations were determined using R2 values displayed in the top right corner.

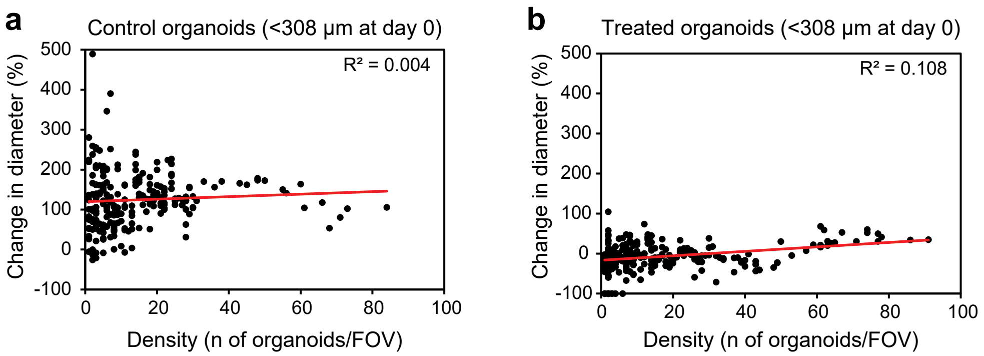

Supplementary Figure S5. Density of the MDCOs does not affect growth or drug response with the change point applied. Scatter plots demonstrate if the density of MDCO cultures affect (a) growth or (b) drug response (n = 3277 and n = 4685, respectively). The change point was applied and only the MDCOs with a starting diameter of <308µm were included for these analyses. For each plot, the average change in diameter per field of view (FOV) was plotted against the number of MDCOs per FOV. Linear trends for each plot are indicated in red. R2 values used to determine correlation are displayed in the top right corner of the plots.

Suppl Figure S6a-b

a

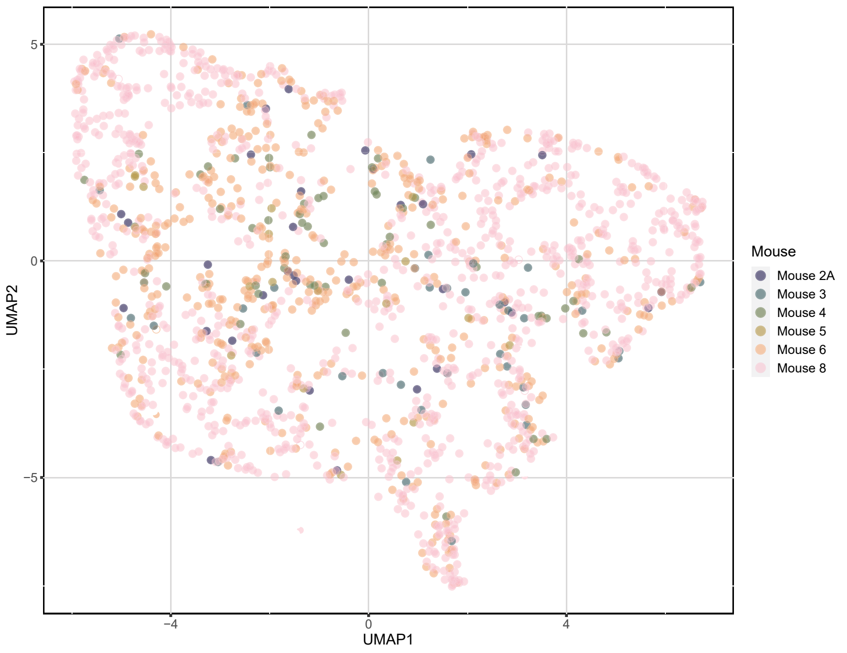

b

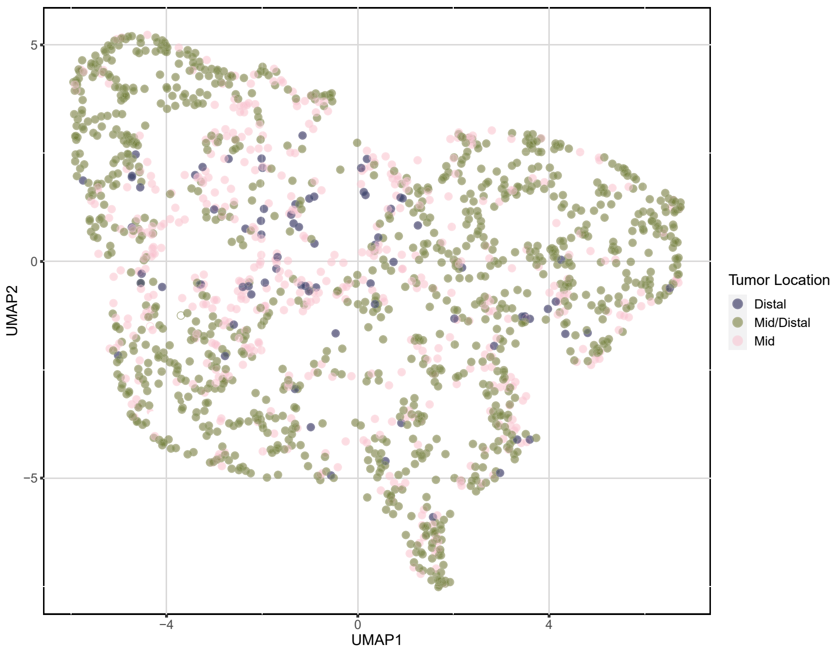

Suppl Figure S6c-d

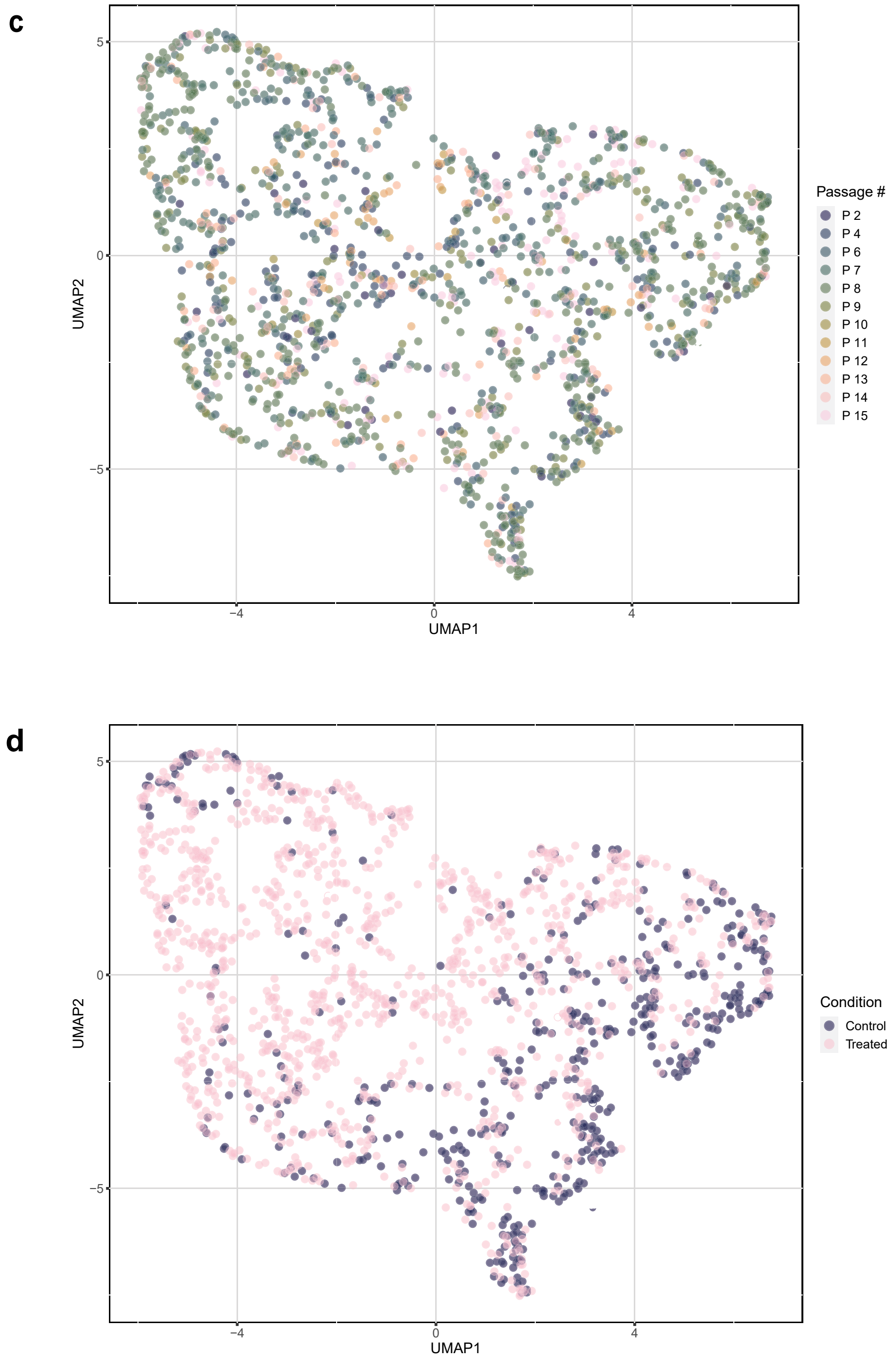

Supplementary Figure S6. UMAP multivariate analyses with change point applied validates that most baseline culture conditions do not affect growth or drug response. (a) Different mice of the same genotype, (b) location of original tumor within the colon, and (c) passage number did not cause any clustering of MDCOs. Only (d) treatment status of an MDCO showed any clustering. (n = 1614 organoids)
